# Design of physical education teaching in colleges and universities based on emotional education under the environment of internet sharing information

**DOI:** 10.3389/fpsyg.2022.1030038

**Published:** 2022-11-11

**Authors:** Wentao Hou, Long Li, Shuying Xing

**Affiliations:** ^1^Department of Physical Education, Soochow University, Suzhou, Jiangsu, China; ^2^Department of Wushu and Art, Nanjing Sport Institute, Nanjing, Jiangsu, China; ^3^Tianjin University of Sport, Tianjin, China

**Keywords:** physical education in colleges and universities, emotional education, information sharing, traditional physical education, design of physical education teaching

## Abstract

In the traditional teaching, the lack of emotional education for students leads to emotional indifference between teachers and students and low teaching quality. In this paper, from the perspective of emotional education. In college physical education teaching, information sharing through the Internet is compared with traditional education in terms of timeliness of communication, integrity of emotional education, teacher-student relationship, and teaching quality. The results show that the timeliness of communication has increased by 9.3%, the integrity of education has increased by 30%, and the relationship between teachers and students has also been significantly improved. In terms of teaching quality, students are more satisfied, and the teaching quality has also been improved. It also shows that emotional education in the Internet sharing information environment can better help students improve their learning, improve their learning efficiency, and learn better in happiness. Emotional education in a shared information environment can better enrich teaching content and enhance students’ enthusiasm, which is of great significance.

## Introduction

**Figure 1:**
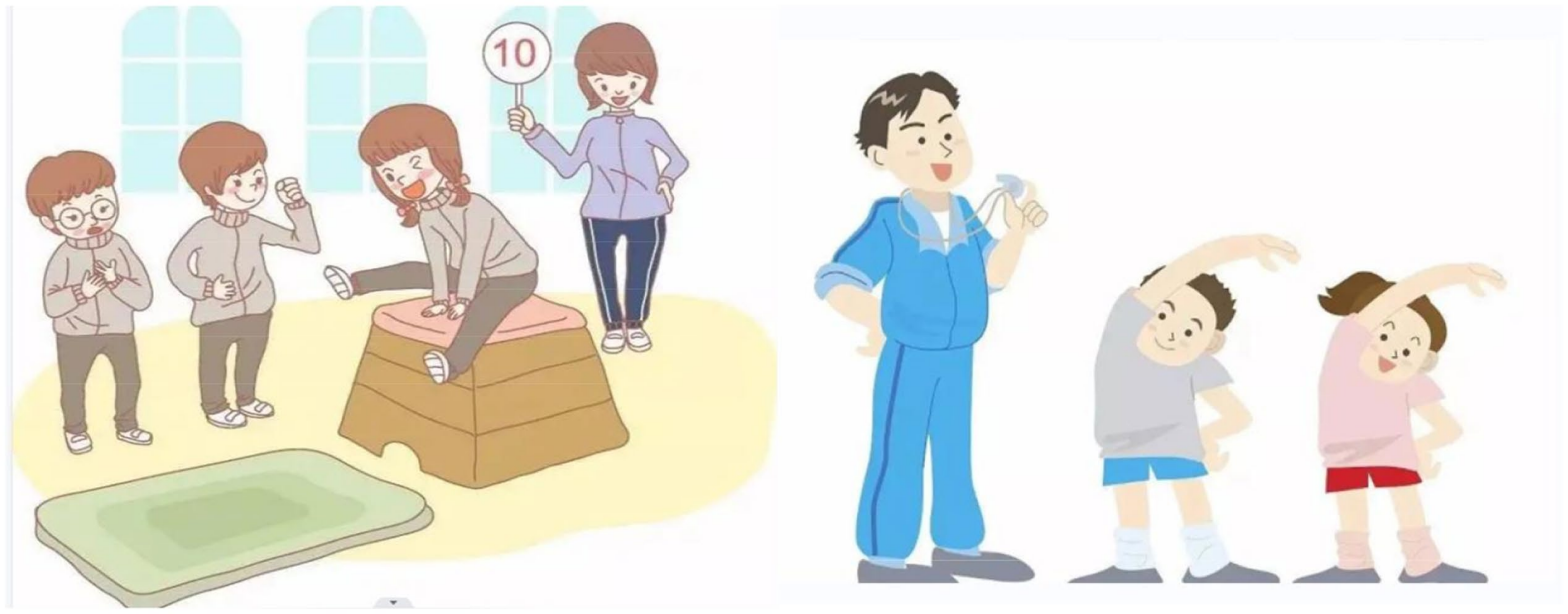
Physical education in colleges and universities.

In the physical education teaching in schools (as shown in Figure 1), physical education teachers as organizations and leaders play a vital role. By setting a teaching topic, students are made to make various movements around the topic, affecting students in emotional ways, which allows them to resonate emotionally with their teachers, thereby actively participating in classroom learning. In teaching practice, students’ emotional education should be based on equality and trust. It is necessary to focus on stimulating students’ interests, activating students’ thinking, satisfying students’ wishes, and creating harmonious classrooms in various forms, so that students can learn and have fun in sports activities.

Emotional education in the environment of sharing information on the network can effectively reduce the tension of students and make it a link between teachers and students. At the same time, it can also make students realize their own subjectivity in the classroom, activate their thinking, and make them better integrate into classroom teaching.

This paper discusses the necessity of implementing emotional education in college physical education, and conducts exploratory research on how to use emotional teaching method. It is necessary to enhance the communication with their families to adapt to their physical and mental needs and create a good educational atmosphere. In addition, it is necessary to be good at exploring educational resources. Through the emotional communication skills between teachers and students, students can form mutual trust and resonate emotionally with classmates. In this way, the enthusiasm of college students is fully mobilized, so as to love the course of physical education, achieving the ideal educational purpose.

In this paper, the data of students majoring in physical education in college teaching is obtained by computer, and the emotional education of campus sports is verified by questionnaire, and the data obtained are classified, analyzed by firefly algorithm, and investigated by questionnaire. It is found that emotional education has a promoting effect on physical education teaching.

## Related work

The 21st century is the era of science and technology. Science and technology not only make people’s lives more convenient, but also promote the progress of civilization, with an impact on the way physical education is taught in colleges and universities. Zhao and Yang studied the application of computer-aided teaching in physical education in colleges and universities. First, the relevant theoretical information and fundamental concepts were introduced. Then combined with physical education, the role of CAI (Computer Assisted Instruction) in this process was discussed, the teaching effect of the computer-aided physical education platform and the traditional teaching method is compared and analyzed. The research results show that the computer-aided physical education platform runs stably, realizes the expected design function, can enrich the teaching content, enhance students’ initiative awareness, stimulate students’ interest in learning, and effectively improve the teaching effect (Zhao and Yang, 2019). With the accelerated development of the information age, how to integrate school sports with information technology has caused a series of problems. The fundamental problem of “students like sports but not in physical education classes” still has not been solved by a better solution. In this regard, Guo et al. takes the positioning of “teaching” and “learning” of college sports empowered by information technology as a logical starting point, from the perspective of conceptual exploration to reality inspection, and analyzes the comprehensive intervention of information technology under the guidance of the “learner-centered” concept. The role and status of “teaching” and “learning” in college sports point out the direction for the integration of school sports and information technology (Guo and Feng, 2021). Wang et al. believe that the lack of effective connection of physical education courses is a prominent problem that hinders the development of physical education in schools. By providing new combination regulations for the structural elements of physical education courses by means of “technical empowerment,” fundamentally change the way of education organization and teaching style (Wang et al., 2021). Zhang and Sun advocates that through big data and informatization means, the whole picture of physical education teaching can be displayed from an overall perspective, and the differences and individualization of physical education teaching can be effectively taken into account an important starting point and effective way of decision-making ability (Zhang and Sun, 2022). Researchers and scholars combined with the development of information technology have provided a unique research perspective for the innovation of physical education teaching in colleges and universities, but the discipline still needs to reposition the relationship between emotional education, information technology, and physical education teaching.

The notion of cognitive teaching, which is integral to all educational activities, stands in opposition to the philosophy of emotional education. In order to improve student emotional communication, cultivate and develop their precious emotions, and mobilize students’ interest and exploration ability, teachers can use emotional teaching, which involves giving full play to the positive power of students’ emotional factors in learning activities. For teachers, they should create a harmonious and harmonious classroom environment that is beneficial to students’ learning emotions, and properly handle the connection between students’ emotions and cognitive abilities, so as to help students develop independent, healthy personalities and personality traits. In response to the formative requirements identified by teachers and students in the district, Zamora G M outlined the systematization of an emotional education curriculum as part of a redesign of the university’s early childhood education curriculum. This course activity was completed by students in their first semester and was aimed at improving self-worth, emotional intelligence, and communication skills to foster positive professional and personal identity growth. His findings shed light on people’s attitudes toward interpersonal, emotional and physical interactions and how they relate to the function of educators, and his experience magnified the importance of emotional education in developing future early childhood educators (Zamora, 2020). Impact assessment of emotional education programs is a related topic that highlights the need to involve participants from different life cycles and settings. In this regard, Castro M evaluated the effects of emotional empowerment programs for women in Mexico. Effects were assessed using the Social–Emotional Skills Scale, the Adult Resilience Scale, and the Anxiety Scale by planning ~10 sessions and considering the results of early and follow-up assessments. His findings showed that family cohesion, personal and social competence, and anxiety were all significantly improved on measures of resilience (Castro et al., 2020). The full and active participation of citizens in the public sphere is the main goal of any thorough democratic education. The development of key cognitive-emotional abilities is key to achieving this goal, but there is no diverse environment to train these abilities. In Read H’s view, actions can be taken to build a more holistic environment where people can learn cognitive-emotional skills and interact positively with others, so that citizens can be actively prepared in their respective fields (Read, 2021). The above scholars have talked about and done research on emotional education, but not in the context of Internet information sharing. In view of this, this paper explores the issue of integrating physical and emotional education in colleges and universities using the online information sharing environment.

## Situation of emotional education in physical education teaching in colleges and universities

### Teaching goals of emotional education

After the establishment of high-quality education, emotional education sets new, more stringent standards for teachers (Yan and Zhang, 2005). First of all, when teaching, teachers must make themselves full of vitality, full of positive emotions, and show their love for the profession of teachers and care for students from the bottom of their hearts. Secondly, emotional teaching requires teachers to communicate emotionally with students in accordance with teaching content and teaching characteristics in order to cultivate students’ emotions. Emotional education refers to education at the emotional level. Educators take corresponding actions according to specific educational needs to promote positive changes in students’ emotions, thereby generating new emotional qualities.

**Figure 2:**
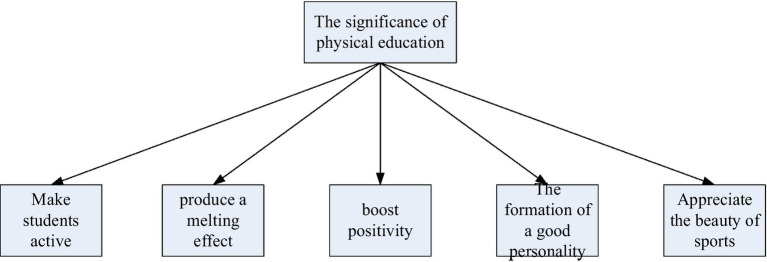
Significance of physical education teaching.

In physical education, emotional education for students can not only mobilize students’ good emotions, but also optimize teaching effect and improve teaching quality (Zach and Rosenblum, 2021). The five functions of physical education are shown in Figure 2.

Physical education is an ongoing and innovative activity, which requires the participation of teachers’ thoughts, emotions, will and other psychological elements. Positive emotions can make them renew their thoughts, free their spirits, and be active in their minds to break out of stereotypes, thus promoting students’ active thinking and students’ creativity.

Emotions can play a role in dissolving, so that students can establish the correct learning motivation, and can also make students more active in sports activities, so as to achieve better curriculum effects (Teraoka et al., 2021).

In physical education, teachers and students are the main body of teaching. Their emotions are expressed through specific facial expressions, body postures, and intonations, which together constitute a signaling system that conveys concepts.

In physical education, by exercising students, they can understand mutual respect, teamwork and other awareness, and cultivate their own independent and positive personality.

In sports activities, the strength, speed, agility, and vitality of the human body are fully displayed, and physical beauty and spiritual beauty are reflected (Gaffney, 2013). Emotional education in physical education can make students really like sports, cultivate students to discover the beauty in sports, and help students to broaden their horizons and enhance their appreciation of sports beauty. Emotional education is not to infuse students with various thoughts and values, but to pay attention to students’ emotions and emotions, so that they can experience happiness and value in physical education. Only in this way can the effect of physical education be maximized, and students can be relieved from the pressure of learning, so as to achieve better learning effect.

### Emotional educational significance of physical education in colleges and universities

**Figure 3:**
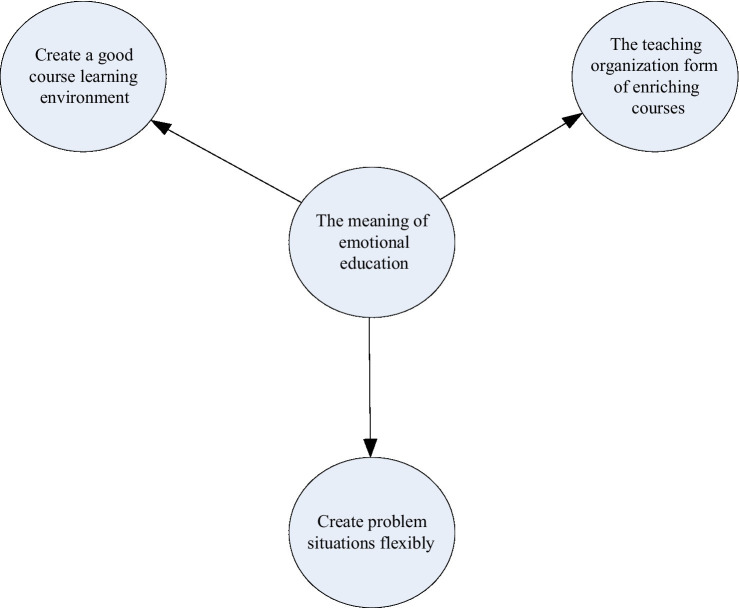
Utility of emotional education.

Emotional education can enrich the way classroom teaching is organized. There are various ways to implement emotional education in physical education curriculum. First of all, it is necessary to be good at enriching the teaching organization form, and make reasonable planning for the teaching process (Wright and Richards, 2022), which can not only improve the teaching effect, but also better integrate the emotional education of students into the classroom. In order to make the teaching content more diverse, a variety of interesting teaching methods can be designed according to different teaching purposes. At the same time, teachers should organize activities that can attract more students to participate as much as possible, which can not only provide each student with a space for activities, but also allow them to experience the joy of games and activities in a good environment. Only in this way, students can have positive emotions and can effectively carry out emotional education. The emotional education significance of physical education is shown in Figure 3.

Teachers can flexibly use teaching strategies such as games and happy learning, and adjust classroom technical operations, so as to allow students to participate in the learning process (Ros-Morente et al., 2022), such as “shooting into the basket,” “throwing sandbags” and so on. Due to different students, with different abilities, there is no guarantee that all students can achieve the same grades. Therefore, when teachers evaluate students’ performance, they must be fair and protect the dignity of students. When teachers conduct teaching evaluation, they should not only enrich the organizational structure of the curriculum, but also consciously teach students according to their aptitude. Only in this way can justice be done, which is also a good emotional education.

Emotional education can flexibly create problem scenarios (Yun and Cho, 2021). In physical education, teachers can also flexibly create problem scenarios according to the teaching content, which not only allows the students to use their brains and make them better think about the relevant actions, but also allows them to better understand and absorb the relevant knowledge. Students experimenting in questioning can make their exploration enthusiasm continuously improve and develop. When teachers explain and correct, students can be more attentive and the effect can be better. Teachers should be good at creating problem environments flexibly. In this way, the teaching effect of knowledge can be improved, and the enthusiasm of students can also be mobilized, so as to achieve positive results and allow students to gain more knowledge in physical education classes.

Emotional education can also create a good learning atmosphere (Zhu and Wang, 2017). Creating a good environment where students can concentrate is also a proven method. Teachers should be adept at flexibly adjusting and changing their curriculum to enliven boring topics and stimulate students’ interest in learning. Additionally, teachers can create engaging study exercises based on the course material to help students understand what they have learned and improve their learning outcomes. At the same time, teachers should take the initiative to participate in students’ learning, where they not only can participate in the competition, but also can serve as a judge of the competition. This can not only shorten the relationship between students and students, but also make the form of the classroom more diverse, thus improving the quality of teaching. In many training contents, if teachers can flexibly change the training method, unexpected effects can often be produced.

### Emotional characteristics of physical education teaching in colleges and universities

**Figure 4:**
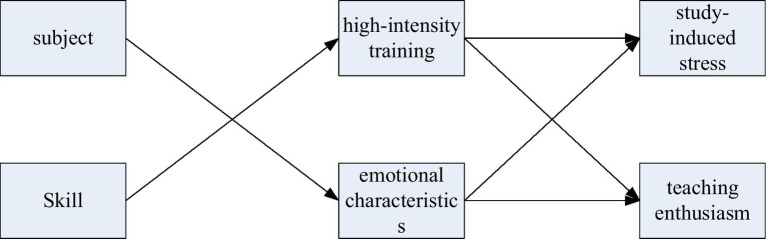
Teaching emotional characteristics.

The characteristics of college sports emotional education seem to be independent of each other, but in fact they influence and restrict each other (Jae et al., 2017), as shown in Figure 4. Therefore, in college physical education, it is necessary to comprehensively analyze the various characteristics of emotional education, but not to grasp each characteristic separately. Physical education should be good at discovering and creating situations, encouraging students with care, teaching by words and deeds, and taking practical actions to stimulate their potential, mobilize their enthusiasm for learning, and enhance their autonomy.

### Design of information sharing platform for emotional education in physical education teaching in colleges and universities

In this section, the first step is to determine the data mode, and connect the data to form the data that users can query through computer access to the database. First of all, we need to collect data through computers, access the sports teaching platform through computers for integration and processing, analyze and process the collected information, and use the firefly algorithm to analyze, verify, and incorporate the post analyzed and processed information into the comprehensive information system.

Data design patterns for information sharing platforms: To build an information sharing platform, the primary task is to select a data model. Currently, the mainstream data models are centralized and distributed (Shao et al., 2018).

Centralized data structure is to build a huge global database, and all data are stored in this database. Then all databases are linked. The data in the database is converted into user-identifiable data so that users can query through this database. The advantage of a centralized data structure is that the data is centralized in one place for easy management and maintenance. However, it requires too much central processing power, which leads to interruption of communication, resulting in the system not working for a long time, and wasting the user’s time and energy.

The distributed data model is to concentrate the data in a single database, and form a unified data sharing platform from different databases. Users can use this database to query the data. The advantages of the distributed data model lie in the strong independence of each business department, high reliability, low communication costs, low investment, and easy cost control, but the management and maintenance of the system are difficult.

Based on the in-depth study of its advantages and disadvantages, this paper proposes a solution using a distributed data model, which makes the use of data more convenient and ensures the security and availability of data. The reason is that the distribution is organized so that the data is distributed on different nodes. If there is a problem with one of the nodes, the data of the other nodes can also run normally and cannot be affected in any way.

The use of database technology has expanded with the development of the network, but there are many changes in the database in the network due to different technologies. Converting heterogeneous data into regular data is the key to building a data sharing platform.

Collection of physical education information resources (Zhang and Zhang, 2022): The construction of the physical education platform is inseparable from a special website. The current physical education platform has low information relevance, and these resources must be classified and integrated in order to include the sports resources in the website, so that physical education teachers and students can clearly find what they want, and upload their materials to the corresponding modules. At the same time, a large amount of information requires website editors to conduct public information processing on the website to classify information, provide sharing and learning functions, and prevent users from wasting time searching for information.

Analyzing and processing information in sports teaching: To establish an efficient sports information integration and sharing platform, the duplication of information should be reduced as much as possible, and its authority and accuracy should be reviewed and verified in order to ensure that the majority of sports information users can obtain effective information more conveniently, comprehensively, professionally and efficiently. A platform provider is an organization that analyzes and processes sports information. For example, athletes who have passed the real-name certification can share their own experience and experience. The platform provider should sort out the uploaded sports knowledge. It needs to review, process and recommend a large amount of sports information to ensure its usability, and also to establish a good personal file, such as the exchange of sports skills, and the editors should have basic sports knowledge, recommend the promotion of sports skills and sportsmanship, and delete those that are not practical.

**Figure 5:**
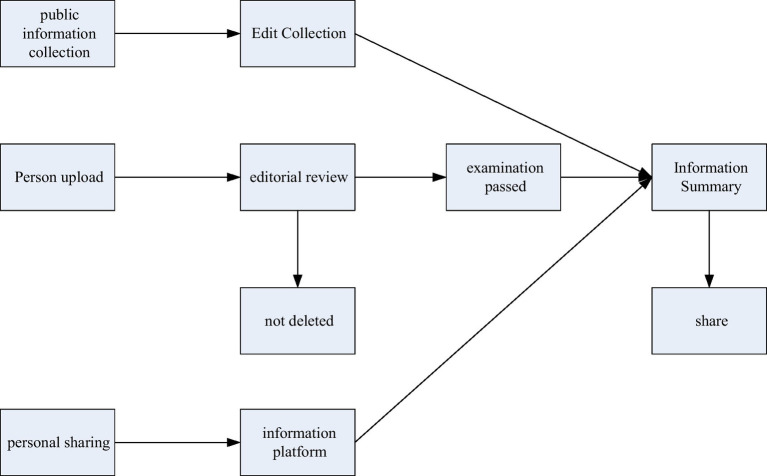
Schematic diagram of the integration of sports information resources.

Comprehensive information system of physical education: The effective integration of physical education information resources can not only promote the maximum sharing of physical education information resources, but also promote the full use of physical education information resources, which can maximize the role of sports information resources, resulting in better social, and economic benefits (Victor et al., 2020). The Internet is a communication platform and an important information transfer station. It can transmit information through people’s communication, such as Weibo, blog, and so on. People can use the Internet to express their ideas and obtain more information. In this specialized website, users are mainly people who are engaged in sports professions, so the resonance of the information is very high. Information integration should be carried out at three levels of disclosure, upload, and sharing. The schematic diagram of the integration of sports information resources is shown in Figure 5.

### Adaptive FA algorithm for information sharing

**Figure 6:**
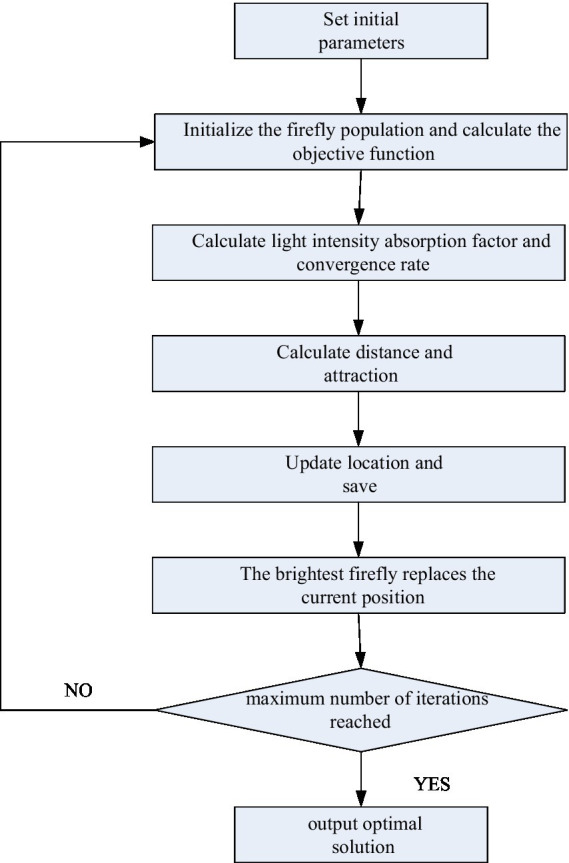
Algorithm flow.

Yang gave the calculation method of FA (Firefly Algorithm) by communicating the light-emitting mode and information of fireflies in nature (Liu and Ye, 2011). Two important influencing factors of the FA method are fluorescence intensity and attractiveness. The main flow chart of the algorithm is shown in Figure 6.

Assuming that the number of fireflies is n and the dimension space is d, then the i-th firefly $x_{i}$ in the d-dimension can be expressed in the following way:


(1) $$x_{i}=x_{i1},x_{i2},..x_{id}$$

Fluorescence brightness is defined as Formula 2:


(2) $$I=I_{0}e^{-r\gamma }$$

In Formula 2, $I_{0}$ is the maximum fluorescence intensity of fireflies, that is, the intrinsic fluorescence brightness of r = 0, which depends on the value of the objective function to be optimized, and is usually expressed by Formula 3. The better f(x), the higher the $I_{0}$ . γ is the light intensity absorption coefficient, which means that the fluorescence intensity changes with the propagation distance and the medium. In practice, a constant of [0.01, 100] is often used. r is the distance between two fireflies, usually expressed by Euclidean distance.


(3) $$I_{0}=f\left(x\right)$$

In Formula 3, f is a function to be optimized, and x is the spatial location of a particular firefly.


(4) $$r_{ij}=x_{i}-x_{j}=\sqrt{\overset{d}{\underset{k=1}{\sum }}x_{i,k}-x_{j,k}}$$

Attractiveness β is defined as Formula 5:


(5) $$\beta =\beta_{0}e^{-\gamma r^{2}}$$

In Formula 5, $\beta_{0}$ is the maximum attraction degree. The distance between fireflies is 0 at this time, and the maximum attraction degree is set to 1 (Li et al., 2022).

Position update formula is as Formula 6:


(6) $$x_{i}\left(t+1\right)=x_{i}\left(t\right)+\beta \left(x_{j}\left(t\right)-x_{i}\left(t\right)\right)+\alpha \epsilon_{i}$$

Formula 6 describes that the firefly i is affected by the firefly j, causing the spatial displacement to change. In Formula 6, t is the iteration time. $x_{i}$ and $x_{j}$ represent the spatial displacement of fireflies i and j, respectively. β is the relative attraction of the power distribution terminal J to the firefly i. α is the step size variable and its interval is [0, 1]. $\epsilon_{i}$ is a random vector obeying a Gaussian or uniform distribution, and its simplified form is rand-1/2. Rand is a random number in [0, 1] that satisfies the average permutation.

The value of γ is recommended to be in [0.01, 100], generally a fixed value of 1. For such a case, it can be seen from the previous attraction that when r is very large, $e^{-\gamma r^{2}}\to 0$ , so that the attraction tends to 0. Substituting into Formula 6, then Formula 7 can be obtained:


(7) $$x_{i}\left(t+1\right)=x_{i}\left(t\right)+\beta \left(x_{j}\left(t\right)-x_{i}\left(t\right)\right)+\alpha \epsilon_{i}\approx x_{i}\left(t\right)+\alpha \epsilon_{i}$$

It can be seen from Formula 7 that the attraction of fireflies to $x_{i}$ is very low. At this time, the firefly becomes a random search for the surrounding environment, and its search effect can only be adjusted through the slight adjustment of $\alpha \epsilon_{i}$ , but cannot communicate between groups. This can reduce the search efficiency of FA.

Update of process information: It can be seen from the steps of the above FA algorithm that in each iteration of the FA algorithm, when the firefly is close to other better fireflies, the FA algorithm only continuously updates the position of firefly $x_{i}$ according to Formula 5, instead of calculating the fluorescence intensity of firefly $x_{i}$ after each position change. That is to say, in the process of searching, the firefly $x_{i}$ constantly changes its position, causing the firefly to move in a better direction. This method not only cannot effectively solve the local search problem of the FA algorithm, but also tends to converge in a short time. To achieve this, in each iteration, the changing positions of the fireflie $x_{i}$ is kept and the corresponding fluorescence intensity is calculated. Every time a firefly is reused, the current firefly is updated in the brightest spot, not the last modified firefly.

According to the evolution law of fireflies in Formula 6, it can be seen that the individual evolution of fireflies mainly depends on the attraction produced by the luminous body. However, due to the limitation of distance, in a certain area, if it exceeds this range, even if there is strong fluorescence, it is difficult to promote the evolution of individuals. Therefore, the FA algorithm fails to make good use of the global information of the population and lacks the attraction to the global, which leads to the application of the algorithm in local optimization. In order to improve the overall search performance of FA, it is difficult to just rely on the above numerical adjustment method, and the evolution of Formula 6 must be improved by introducing other global information.

Assuming that the FA system is a quantum system with an attractor P, and the instantaneous state of the system is represented by the wave function Ψ , ${\left|\Psi \right|}^{2}$ represents the probability density function at the location point. The wave function in generation (t + 1) is as Formula 8:


(8) $$\Psi \left(x_{\mathrm{id}}\left(t+1\right)\right)=\frac{1}{\sqrt{L_{\mathrm{id}}\left(t\right)}}e^{-\left|x_{\mathrm{id}}\left(t+1\right)-p_{\mathrm{id}}\left(t\right)\right|/L_{\mathrm{id}}\left(t\right)}$$

The probability density function Q corresponding to individual i is as Formula 9:


(9) $$Q=\frac{1}{L_{\mathrm{id}}\left(t\right)}e^{-2\left|x_{\mathrm{id}}\left(t+1\right)-p_{\mathrm{id}}\left(t\right)\right|/L_{\mathrm{id}}\left(t\right)}={\left|\Psi \left(x_{\mathrm{id}}\left(t+1\right)\right)\right|}^{2}$$

Then the probability distribution function F of individual i is as Formula 10:


(10) $$F\left(x_{\mathrm{id}}\left(t+1\right)\right)=e^{-2\left|x_{\mathrm{id}}\left(t+1\right)-p_{\mathrm{id}}\left(t\right)\right|/L_{\mathrm{id}}\left(t\right)}$$

In Formulas 8–10, i is the number of a single firefly, and d is the dimension. t is the number of iterations. $p_{\mathrm{id}}$ is the attractor, which is also called the attraction position. $L_{\mathrm{id}}\left(t\right)$ is the characteristic length. By means of random simulation, the individual is measured, and the position of the (t + 1)-th generation firefly individual i on the d-dimension can be obtained:


(11) $$x_{id}\left(t+1\right)=p_{id}\left(t\right)\pm \frac{L_{id}\left(t\right)}{2}\ln\left(1/\mu \right)$$

In Formula 11, μ is a random number uniformly distributed between 0 and 1. When rand(0, 1) > 0.5, the sign “+” is taken; otherwise the sign “−” is taken. The value of $L_{\mathrm{id}}\left(t\right)$ is determined by Formula 12:


(12) $$L_{\mathrm{id}}\left(t\right)=2\tau \left|c_{d}\left(t\right)-x_{\mathrm{id}}\left(t\right)\right|$$

In Formula 12, $c_{d}\left(t\right)$ is the average position information of the t-th generation firefly population in the d-dimension. τ is the contraction-expansion factor, which can be converged by adjusting the value.


(13) $$c\left(t\right)=\frac{1}{n}\overset{n}{\underset{i=1}{\sum }}x_{i}\left(t\right)$$

(14) τ=1−t/maxG∗0.5

Among them, n is the group size, and maxG is the maximum number of iterations.

The FA method is to transfer the fireflies with lower fluorescence height or the fireflies with higher fluorescence height, and move them into the power distribution terminal with higher fluorescence degree, and then move them into the optimal individual. This method is dynamically generated by Formula 6. Since the fireflies in each dimension are relatively independent individuals, they can be subscripted in the dimension. A brand new location update mode is as Formula 15:


(15) $$\begin{array}{c} x_{i}\left(t+1\right)=x_{i}\left(t\right)+\beta \left(x_{j}\left(t\right)-x_{i}\left(t\right)\right)\\ \pm \tau \left|c\left(t\right)-x_{i}\left(t\right)\right|*\ln\left(1/\mu \right)+\alpha \epsilon_{i} \end{array}$$

The exchange of data information is explained by obtaining the position information of fireflies.

## Teaching in a sharing information environment compared with traditional teaching

A random questionnaire survey was conducted on 200 students in two different colleges and universities, and the questionnaire recovery rate was 100%. The number of experiments was three. Through the comparison of teaching in the shared information environment (referred to as new teaching in figures) and traditional teaching, the comparison was mainly made in four aspects: the timeliness of communication between the two, the integrity of emotional education, the relationship between teachers and students, and the quality of teaching. The teacher-student relationship was judged according to the students’ responses to the questionnaire. Through the analysis of the students’ responses in the four stages of poor, average, good, and very good relationship with teachers, the quality of teaching was also scored according to students’ feelings.

**Figure 7:**
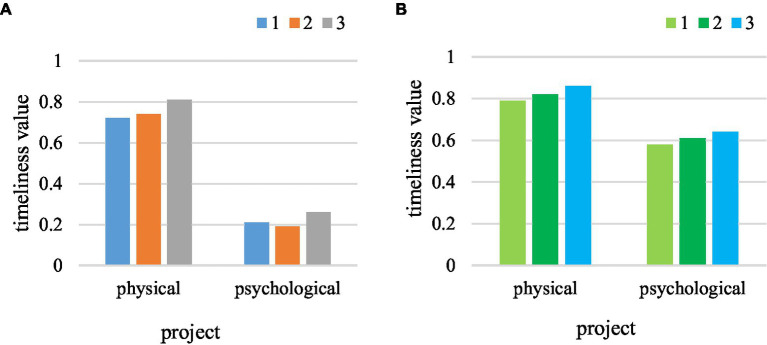
Comparison of timeliness. **(A)** Traditional teaching. **(B)** New teaching.

In physical education, the timeliness of communication with students is very important for regulating students’ emotions and states, which is related to students’ evaluation of teaching quality and students’ attitude toward teaching. The timeliness of communication with students under the two teaching modes is shown in Figure 7. The range of timeliness in Figure 7 is between 0 and 1. The larger the value, the higher the timeliness.

As can be seen from Figure 7, the average timeliness of students’ physical problems in traditional teaching was 0.75, but the timeliness of psychological problems was only 0.22. In the new type of teaching, the timeliness of students’ questions has been improved, reaching 0.82, with a relative increase of 9.3%. The average psychological timeliness reached 0.61, with an increase of 0.39, and had a significant improvement. It showed that in the environment of sharing information on the Internet, great attention has been paid to the timeliness of students’ psychology and emotion.

**Figure 8:**
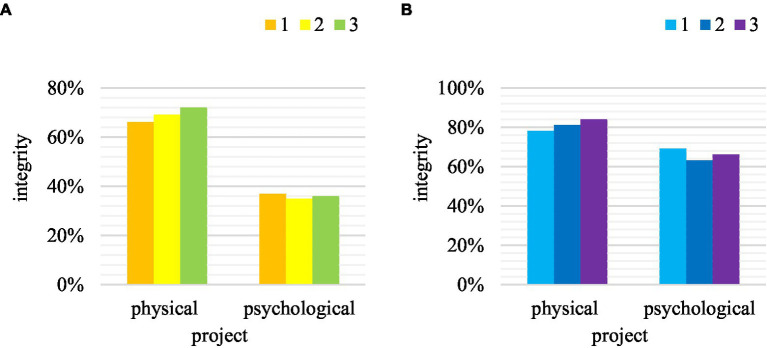
Comparison of teaching integrity. **(A)** Traditional teaching. **(B)** New teaching.

The information of traditional physical education is not complete, all relying on teachers’ own knowledge to teach students. In the case of sharing information in the Internet of Things, the integrity of the teaching information can be more complete. The comparison between the two in the completeness of teaching information is shown in Figure 8.

From the two sets of data charts, it can be found that the content integrity of traditional physical education in emotional teaching was only 36%, but in the new type of teaching, the integrity reached 66%, with an increase of 30%. The completeness of physical knowledge in physical education has increased from 69% to 81%. It also showed that in the case of information sharing, the overall teaching content of physical education has been greatly improved.

**Figure 9:**
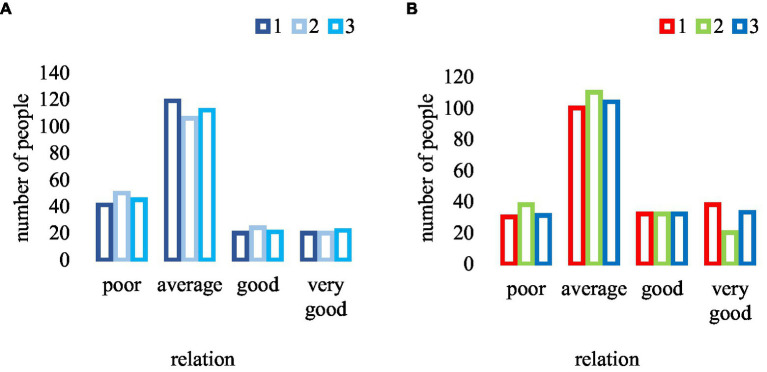
Comparison of teacher-student relationship. **(A)** Traditional teaching. **(B)** New teaching.

The teacher-student relationship plays an important role in teaching and also affects all aspects of physical education. Especially emotionally, in the process of mutual contact between students and teachers, the harmonious relationship between students and teachers is also conducive to teaching. The comparison chart of the teacher-student relationship between traditional teaching and new teaching is shown in Figure 9.

From the data in Figure 9, it can be found that among the number of students who evaluate the relationship with the teacher, in traditional teaching, 22.6% had a bad relationship with the teacher, 56.1% with an average relationship, 10.8% with a good relationship, and 10.5% with a very good relationship, but in the new type of teaching, 16.5% had a bad relationship, 52.3% with an average relationship, 16% with a good relationship, and 15.2% with a very good relationship. It can be found that under the new type of physical education, the number of people with poor relationships decreased by 6.1%, while the number of people with good relationships increased by 5.2%, and the number of people with good relationships increased by 4.7%. From these data, it can also be explained that in the case of new physical education, it helped to build a bridge of communication between students and teachers, and could better help students solve more problems.

**Figure 10:**
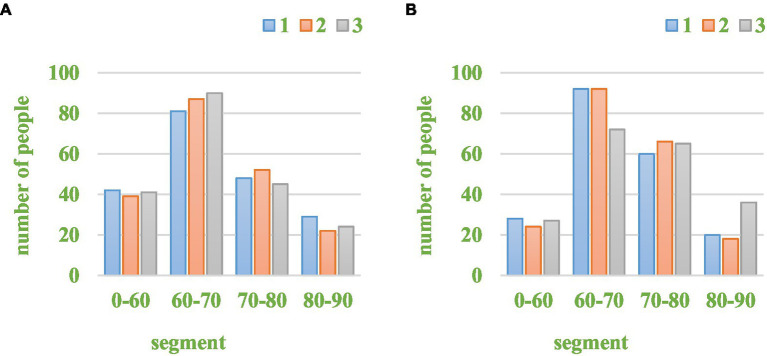
Comparison of teaching quality scores. **(A)** Traditional teaching. **(B)** New teaching.

For colleges and universities, the quality of teaching is the most important part. If there is a problem, the management education of the integrated school can be affected. Students evaluated the quality of teaching based on how they feel about the class. Figure 10 shows a comparison of the information from the questionnaire to the teaching quality rating.

As can be seen from Figure 10, the number of people with a score of 0–60 has been reduced, with a relative reduction of 35.2%. The number of people in the 60–70 segment did not change much across the 3 experiments. The number of people in the 70–80 segment increased by 31.2%. The change in the 80–90 segment was not large, and the score did not change much, which indicated that the ratings given were fairly accurate. In the 0–60 segment and the 70–80 segment, distinctions were made. It also showed that in the new type of teaching, the overall teaching quality has been improved, and students had a certain recognition of physical education teaching.

## Discussion

By comparing the timeliness of communication, the integrity of emotional education, the quality of teaching, and the relationship between teachers and students between traditional teaching and information sharing teaching, it is found that the teaching under information sharing is more conducive to improving the quality of teaching and the relationship between teachers and students. In dealing with problems, the timeliness of communication between teachers and students can help students to enhance their trust in teachers as much as possible, which is conducive to teaching and establishing a good image of teachers in students. The integrity of teaching can fully compensate for the emotional vacancy of students, and can also improve the overall teaching quality, which has some important role in promoting college teaching.

## Conclusion

The traditional emotional education in physical education in colleges and universities is deficient. The traditional education lacks the understanding of students’ psychological feelings. Students are prone to conflict with teachers, which affects learning and forms shackles on students’ thoughts. In this paper, the traditional emotional education is designed and studied by introducing Internet sharing information. Mainly in the two aspects of timeliness of communication, integrity of emotional education, teacher-student relationship, and teaching quality. The results showed that the teaching quality was improved in terms of the timeliness of students’ physical problems. It also shows that the new type of teaching will be more in line with the current society, and will also pay more attention to the emotional education of sports students, more promote students’ development of thinking, and truly make students feel happy in sports teaching, which is the original intention of education, happy learning and happy life. The inadequacy of this paper is that the principle of the established platform is not explained, and the technology of sharing information is not explained. For the emotional education of students, we can study it in more detail, such as students’ attendance, positive speech, teacher-student interaction, etc., to better understand the impact of emotional education on students. Internet sharing information can not only be used in teaching, but also in other aspects, such as network security, aerospace, national construction, etc., and plays an unexpected role in more fields.

## Data availability statement

The original contributions presented in the study are included in the article/supplementary material, further inquiries can be directed to the corresponding author.

## Author contributions

WH did the overall design and paper writing for this work. LL provided ideas and help for later revisions of the work. SX guided the technical work of this paper. All authors contributed to the article and approved the submitted version.

## Conflict of interest

The authors declare that the research was conducted in the absence of any commercial or financial relationships that could be construed as a potential conflict of interest.

## Publisher’s note

All claims expressed in this article are solely those of the authors and do not necessarily represent those of their affiliated organizations, or those of the publisher, the editors and the reviewers. Any product that may be evaluated in this article, or claim that may be made by its manufacturer, is not guaranteed or endorsed by the publisher.
